# *Pinellia ternata* attenuates carotid artery intimal hyperplasia and increases endothelial progenitor cell activity via the PI3K/Akt signalling pathway in wire-injured rats

**DOI:** 10.1080/13880209.2020.1845748

**Published:** 2020-11-30

**Authors:** Hai-Ke Lu, Yan Huang, Xiao-Yu Liang, Ying-Yi Dai, Xin-Tong Liu

**Affiliations:** Department of Neurology, Guangdong Second Provincial General Hospital, Guangzhou, China

**Keywords:** Atherosclerosis, EPCs, blood lipids, inflammatory response

## Abstract

**Context:**

Clinically, *Pinellia ternata* (Thunb.) Breit. (Araceae) (*PT*) has been widely used in the treatment of atherosclerosis and hyperlipidaemia, but the underlying mechanisms are still not clearly understood.

**Objective:**

This research was conducted to confirm the mechanism by which *PT* affects carotid artery intimal hyperplasia.

**Materials and methods:**

An intestinal hyperplasia Sprague-Dawley rat model was established by carotid artery injury. The rats were randomly divided into five groups (*n* = 8): sham, model, *PT* (with daily intragastric administration of 10 g/mL/kg *PT* tubers water extract), *PT*+LY294002 (with intraperitoneal injection of 50 mg/kg LY294002 + 10 g/mL/kg *PT*) and endothelial progenitor cells (EPCs) (with injection of 5 × 10^5^/cells), and treated for 4 or 8 weeks.

**Results:**

HE staining showed that *PT* attenuated intimal hyperplasia. RT-PCR, Western blotting and immunohistochemistry showed that *PT* increased the expression of vascular endothelial growth factor (VEGF) and eNOS in the atherosclerotic carotid artery. *PT* increased the Dil-acLDL^+^/FITC-UEA-1^+^ population (from 0.41 ± 0.085% to 0.60 ± 0.092%) in the blood, decreased TCHO, TG, LDL-C, IL-6 and TNF-α levels, and increased HDL-C and IL-10 levels in the blood. However, these changes were reversed by the PI3K/Akt pathway inhibitor LY294002.

**Discussion and conclusions:**

*PT* can be developed as an atherosclerosis and carotid intimal hyperplasia treatment drug. Therefore, further study will focus on the effects of *PT* on intimal hyperplasia in wire-injured atherosclerosis patients and explore in depth some other relevant molecular mechanisms.

## Introduction

Atherosclerosis, the leading cause of death worldwide, is a chronic progressive inflammatory vascular disease. The major clinical outcomes of atherosclerosis are usually related to the disruption of vulnerable atherosclerotic plaques, which leads to thrombosis and embolism (Eldika et al. 2004). In the clinic, endarterectomy for atherosclerosis can cause damage to the vascular endothelium and proliferative restenosis in the arterial blood vessels (Young et al. 2013). Repair after vascular injury directly affects the endarterectomy outcome. The hyperplastic response therapeutic strategy is effective in animal experiments, but the interventions tested thus far have not been shown to have sufficient clinical utility (Lefkovits and Topol 1997). Therefore, further study of the potential molecular mechanisms of the hyperplastic response and continued development of new treatments for intimal hyperplasia restenosis are urgently needed. Yoshimura et al. (2001) noted that NF-κB might be a new therapeutic target for inhibition of neointimal hyperplasia after angioplasty and that inhibition of NF-κB could inhibit neointimal formation after wire injury. In a rat model, neutralizing ICAM-1 antibodies reduced intimal hyperplasia after wire injury of the carotid artery (Yasukawa et al. 1997).

*Pinellia ternata* (Thunb.) Breit. (Araceae) (*PT*), a traditional Chinese medicine, has been widely used to prevent/treat excessive phlegm and emesis (Zhang et al. 2016). Numerous studies have reported that *PT* and its ingredients also have other applications and functions. For example, Yu et al. (2015) found that *PT* lectin has proinflammatory activity and is involved in ROS overproduction, NF-κB pathway activation and subsequent induction of cytokine release and neutrophil migration. A polysaccharide from the tubers of *PT* can inhibit the proliferation of human cholangiocarcinoma cell lines, and this polysaccharide may be able to be developed as a promising drug for the prevention and treatment of human cholangiocarcinoma (Li et al. 2016). Chen et al. (2014) found that tangerine peels and *PT* tubers can upregulate the levels of PBK and p-Akt in vascular endothelial cells (ECs) and play important roles in the treatment of carotid atherosclerosis. Guo et al. (2017) found that Gualou Xiebai Banxia decoction has a significant therapeutic effect on atherosclerotic Apo-E^–/–^ mouse lesions. In the clinic, *PT* has been widely used for the treatment of atherosclerosis and hyperlipidaemia (Wang et al. 2008).

Despite the increase in the clinical use of *PT*, the mechanisms underlying its therapeutic effects on atherosclerosis are still not clearly understood. In this study, we first established an intimal hyperplasia model and then administered *PT* by gavage to ultimately reveal the underlying mechanisms of the therapeutic effects of *PT* on intimal hyperplasia in the wire-injured carotid arteries of atherosclerotic rats.

## Materials and methods

### Primary reagents and chemicals

*PT* was identified and donated by Mr. Zhiyuan Li from the Guangzhou University of Traditional Chinese Medicine. A voucher specimen was deposited in the sample room of the Guangdong Second Provincial General Hospital (voucher no. PT-201602). LY294002 was purchased from Sigma (cat. no. L9908, St. Louis, MO). A total of 200 g tubers of *PT* was crushed and decocted with 2 L of boiling water for 2 h and filtered. Then, 1 L of distilled water was added, and the mixture was decocted at 100 °C for 1 h and filtered again. The combined water extract was considered the *PT* water extract.

### Establishment of an intimal hyperplasia model through carotid artery injury in atherosclerotic rats

Sprague-Dawley rats (180–200 g, *n* = 80, 6 weeks of age) were purchased from the Experimental Animal Center of Sun Yat-sen University. The rats were fed and watered freely and housed under a constant temperature of 22 ± 2 °C, a humidity of 50–60% and a 12 h light/dark cycle. The animal experiment was approved by the Institutional Animal Care and Use Committee of the Guangdong Second Provincial General Hospital (no. 2016-KYLL-021). Partial ligation of the left common carotid artery (CCA) was performed as previously described (Hoffmann and Mintz 2000). Briefly, anaesthesia was induced by 2% (v/v) isoflurane inhalation. After removing the hair, the neck was disinfected with 75% alcohol, and then a 4–5-mm ventral midline incision was made. The two carotid arteries were exposed by blunt dissection. The right CCA was not ligated and served as an internal control. While the superior thyroid artery was left intact, three of the four caudal branches of the left CCA were ligated with a 6.0 silk suture. The proximal CCA and the distal end of the internal carotid artery were clamped with a microscopic artery clip, and a transverse incision was made in the external carotid artery. Then, a 0.035-inch guide wire was passed through this incision into the CCA and pushed and pulled along the wall of the artery six times. Then, the guide wire was withdrawn, the external carotid artery incision was sutured, and the arterial clip was removed. After model establishment, the experimental rats were fed a high-fat diet for 8 weeks (2% cholesterol and 10.0% fat), while the control rats were fed a regular diet. All efforts were made to reduce the number of rats used and to minimize rat suffering.

### Endothelial progenitor cells (EPCs) preparation and identification

EPCs were isolated from the peripheral blood of Sprague-Dawley rat (180–200 g, *n* = 1, 6 weeks of age) (Zoldhelyi et al. 2001). Briefly, rats were injected intraperitoneally with 30 mg/kg 1% sodium phenobarbital and anaesthetized, and then 5 mL of peripheral blood was obtained from the femoral artery. The blood was added to a tube with an equal volume of lymphocyte separation solution (Haoyang, cat. no. LTS1077, Tianjin, China), and the mixture was then centrifuged two times at 2000 rpm for 10 min. The mononuclear cells were isolated and resuspended in Iscove's modified Dulbecco's medium supplemented with foetal bovine serum (Gibco, Cat. No. 10099-141, Carlsbad, CA), vascular endothelial growth factor (VEGF) (Sigma, cat. no. SRP6020, St. Louis, MO) and basic fibroblast growth factor (bFGF) (Sigma, cat. no. SRP4039, St. Louis, MO) in fibronectin-coated 24-well plates (BD, BioCoat, cat. no. 354144, Franklin Lakes, NJ) in an incubator at 37 °C under 5% CO_2_.

EPCs were identified by flow cytometry using Dil-labelled acetylated low-density lipoprotein (acLDL) and FITC-labelled *Ulex europaeus* lectin-1 (UEA-1). The double-positive cells (Dil-acLDL^+^/FITC-UEA-1^+^) were identified as EPCs (Vasa et al. 2001). Briefly, the cells were collected and resuspended, Dil-acLDL and FITC-UEA-1 were added, and the cells were incubated for 30 min. Finally, the proportion of double-positive cells was examined by flow cytometry (BD, Accuri C6, Franklin Lakes, NJ). The purity of the EPCs exceeded 90% (Supplementary Figure 1) and could thus be used for subsequent experiments.

### Grouping and treatment

Six-week-old Sprague-Dawley rats were randomly divided into five groups (*n* = 8): sham, model (which received intragastric administration of an equal volume of saline), *PT* (which received daily intragastric administration of 10 g/mL/kg *PT* water extract after 30 min of modelling until the time of sacrifice), *PT*+LY294002 (which received 50 mg/kg LY294002 by intraperitoneal injection 1, 2 and 3 days before modelling and half an hour after modelling in addition to *PT* water extract administration as described above) and EPCs (which was injected with 1 mL of a suspension of EPCs cultured *in vitro* at a concentration of 5 × 10^5^/mL via tail vein). The mental state, diet and activity of the rats were observed, and there were no abnormal changes among the groups. At 4 weeks and 8 weeks, the rats were anaesthetized using 1% sodium pentobarbital (40 mg/kg per Sprague-Dawley rat). Celiac venous blood was immediately isolated, and the rats were then sacrificed by excessive anaesthesia. Finally, the CCA and aorta were carefully excised.

### HE staining

The CCA tissues were fixed in 4% (w/v) paraformaldehyde for 20 min and then sliced into 3 μm sections after paraffin embedding. The sections were processed as follows: dewaxed in 70% (v/v) ethyl alcohol for 10 s, washed in diethylpyrocarbonate-treated water for 5 s, treated with haematoxylin with RNase inhibitor for 30 s, washed in 70% (v/v) ethyl alcohol for 30 s, stained in eosin Y for 20 s, dehydrated through a series of alcohol solutions for 30 s each and treated with a xylene solution for 2 min. After sealing the sections with resin, visual analysis was performed with an Olympus inverted microscope (Olympus, CX71, Tokyo, Japan).

### Immunohistochemistry

CCA tissue was cut into 4-mm frozen sections. Following deparaffinization, hydration and blocking with 10% goat serum, the slides were treated with peroxide (Invitrogen, Carlsbad, CA) and incubated with anti-VEGF (Abcam, cat. no. ab32152, Burlingame, CA) and anti-endothelial nitric oxide (NO) synthase (eNOS) (Abcam, cat. no. ab119292, Burlingame, CA) primary antibodies overnight at 4 °C. After being washed in PBS and incubated with HRP-labelled secondary antibodies for 30 min at 37 °C, the slides were stained in a 3,30-diaminobenzidine solution for 2 min. Finally, the slides were lightly counterstained using haematoxylin, dehydrated using ethyl alcohol, and mounted using neutral resin. Five random visual fields were quantified at ×400 magnification, and the mean optical density (MOD) was calculated using Image-Pro Plus 6.0 software (Media Cybernetics, Rockville, MD).

### Quantitative reverse transcription polymerase chain reaction (qRT-PCR)

Using RNA Isolater Total RNA Extraction Reagent (Vazyme, cat. no. R401-01, Nanjing, China), we extracted the total RNA from CCA tissues and then reverse-transcribed it into cDNA. qRT-PCR was performed with AceQ qPCR SYBR Green Master Mix (Vazyme, cat. no. Q111-02, Nanjing, China) using a 7500 Real-Time PCR System (Applied Biosystems, Foster City, CA). The GAPDH gene was used for normalization. The qRT-PCR primers were as follows: VEGF, 5′-CGGGCCTCTGAAACCATGAA-3′ and 5′-GCTTTCTGCTCCCCTTCTGT-3′; eNOS, 5′-AAGTGGGCAGCATCACCTAC-3′ and 5′-GCCGGCTCTGTAACTTCCTT-3′; and GAPDH, 5′-AGACAGCCGCATCTTCTTGT-3′ and 5′-TGATGGCAACAATGTCCACT-3′.

### Western blot analysis

Total proteins were prepared from CCA tissues. The proteins were analysed with 12% SDS polyacrylamide gel electrophoresis. After electrophoresis, the proteins were electrotransferred onto PVDF membranes. Immunoblotting was performed via incubation with anti-VEGF (Abcam, cat. no. ab32152, Burlingame, CA) and anti-eNOS (Abcam, cat. no. ab119292, Burlingame, CA) antibodies at 37 °C for 2 h followed by incubation with goat anti-rabbit HRP-labelled IgG. The protein bands were scanned and quantified using ImageJ 2× software (National Institutes of Health, Bethesda, MD).

### Flow cytometry

Approximately, 1 mL of blood was diluted with D-Hank’s solution. A 1.5-fold volume of Ficoll-Hypaque (Sigma, St. Louis, MO) was added, the mixture was centrifuged at 1200×*g* for 30 min, and the middle layer of cells was obtained. After being washed again with D-Hank's solution, the cells were resuspended in M199 cell medium, and Dil-acLDL and FITC-UEA (Molecular Probes, Beijing, China) were added. After incubating the cells at room temperature for 30 min and then washing them two times in PBS, the proportion of double-positive cells was examined by flow cytometry (BD, Accuri C6, Franklin Lakes, NJ).

### Detection of blood lipids and cytokines

TCHO, TG, HDL-C and LDL-C were detected in the blood using biochemical detection kits (Jiancheng, Nanjing, China) according to the manufacturer’s instructions. The levels of interleukin-6 (IL-6) (Abcam, cat. no. ab100772, Burlingame, CA), interleukin-10 (IL-10) (Abcam, cat. no. ab100765, Burlingame, CA) and tumour necrosis factor-α (TNF-α) (Abcam, cat. no. ab100785, Burlingame, CA) secreted into the blood were assessed with ELISA kits. The absorbance (optical density, OD) was measured at a wavelength of 450 nm.

### Statistical analysis

The statistical analyses were performed with SPSS 19.0 software (IBM Corp., Armonk, NY). The data are presented as the means ± standard deviations (SDs). Significance was determined by one-way analysis of variance with the least significant difference *post hoc* test (when equal variances were assumed) or Dunnett's T3 *post hoc* test (when equal variances were not assumed) or by Student’s *t*-test, and *p* < 0.05 indicated a statistically significant difference.

## Results

### PT inhibits carotid artery intimal hyperplasia after wire injury

**Figure 1.**
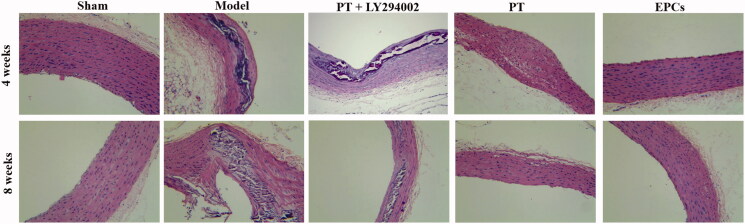
Effect of *PT* on intimal hyperplasia in atherosclerotic rats at 4 weeks and 8 weeks, as determined by HE staining. Magnification: ×200.

In the sham group, the intima of the carotid artery was intact, and the middle membrane contained a large number of spindle-shaped smooth muscle cells. The model group presented obvious intimal thickening and atherosclerotic plaques, but such changes were attenuated in both the *PT* and EPC groups. Conversely, the addition of LY294002 treatment to *PT* treatment weakened the improvement (Figure 1). HE staining indicated that *PT* inhibited intimal hyperplasia of atherosclerotic carotid arteries via the PI3K/Akt pathway.

### PT induces VEGF and eNOS expression in wire-injured rat carotid arteries via the PI3K/Akt pathway

**Figure 2.**
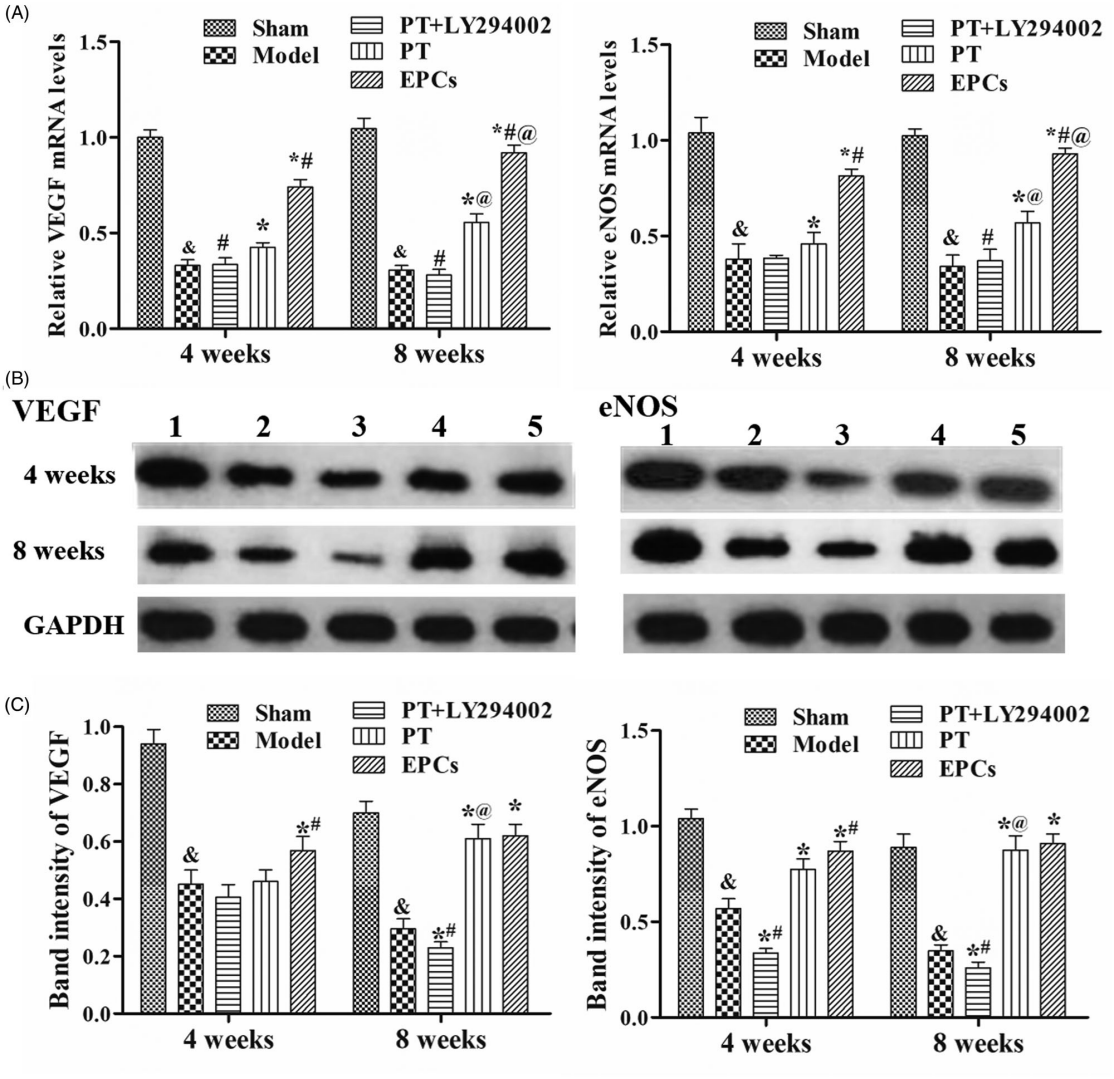
Effects of *PT* on VEGF and eNOS expression levels at 4 weeks and 8 weeks. (A) Relative levels of VEGF and eNOS mRNA in each group. (B) Western blot images of VEGF and eNOS. (1) Sham group, (2) model group, (3) *PT*+LY294002 group, (4) *PT* group and (5) EPC group. (C) The relative expression levels of VEGF and eNOS were calculated via normalization to the levels of GAPDH. The data are presented as the mean ± SD. At 4 weeks and 8 weeks: compared to the sham group, &*p* < 0.05; compared to the model group, *p < 0.05; compared to the *PT* group, #*p* < 0.05; compared to the corresponding 4-week group, @*p* < 0.05.

**Figure 3.**
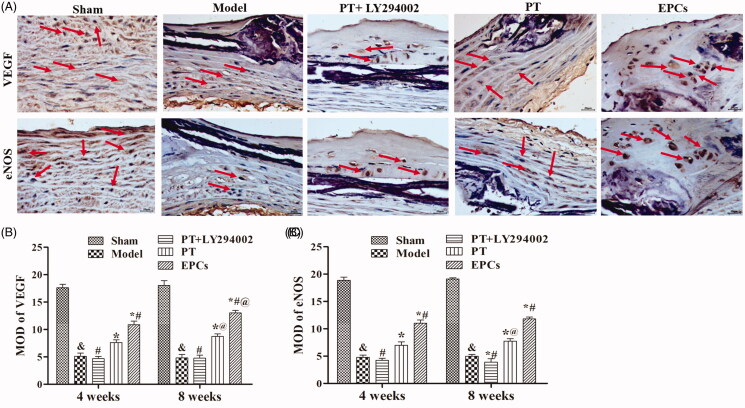
Effect of *PT* on VEGF and eNOS expression as determined by immunohistochemistry at 4 weeks and 8 weeks. (A) Immunohistochemical images of VEGF and eNOS at 8 weeks. (B) The MOD of VEGF at 4 weeks and 8 weeks was calculated using Image-Pro Plus 6.0 software. (C) The MOD of eNOS at 4 weeks and 8 weeks was calculated using Image-Pro Plus 6.0 software. The data are presented as the mean ± SD (*n* = 8). At 4 weeks and 8 weeks: compared to the sham group, &*p* < 0.05; compared to the model group, *p < 0.05; compared to the *PT* group, #*p* < 0.05; compared to the corresponding 4-week group, @*p* < 0.05.

qRT-PCR, Western blotting and immunohistochemistry were used to analyse the expression levels of VEGF and eNOS at various treatment times. As shown in Figures 2 and 3, after the rats were treated for 4 or 8 weeks, the mRNA and protein levels of VEGF and eNOS were both significantly higher in the rats treated with *PT* or EPCs relative to the rats in the model group (*p* < 0.05), and the *PT*+LY294002 group did not exhibit higher protein and mRNA expression levels of VEGF and eNOS than the *PT* group. In addition, LY294002 treatment inhibited the activation of the PI3K/Akt pathway (decreased PI3K and phosphorylated Akt (p-Akt) expression) (Supplementary Figure 2). These results indicate that *PT* induces VEGF and eNOS expression in atherosclerotic carotid arteries via the PI3K/Akt pathway.

### PT increases the EPC proportion in wire-injured carotid arteries via the PI3K/Akt pathway

**Figure 4.**
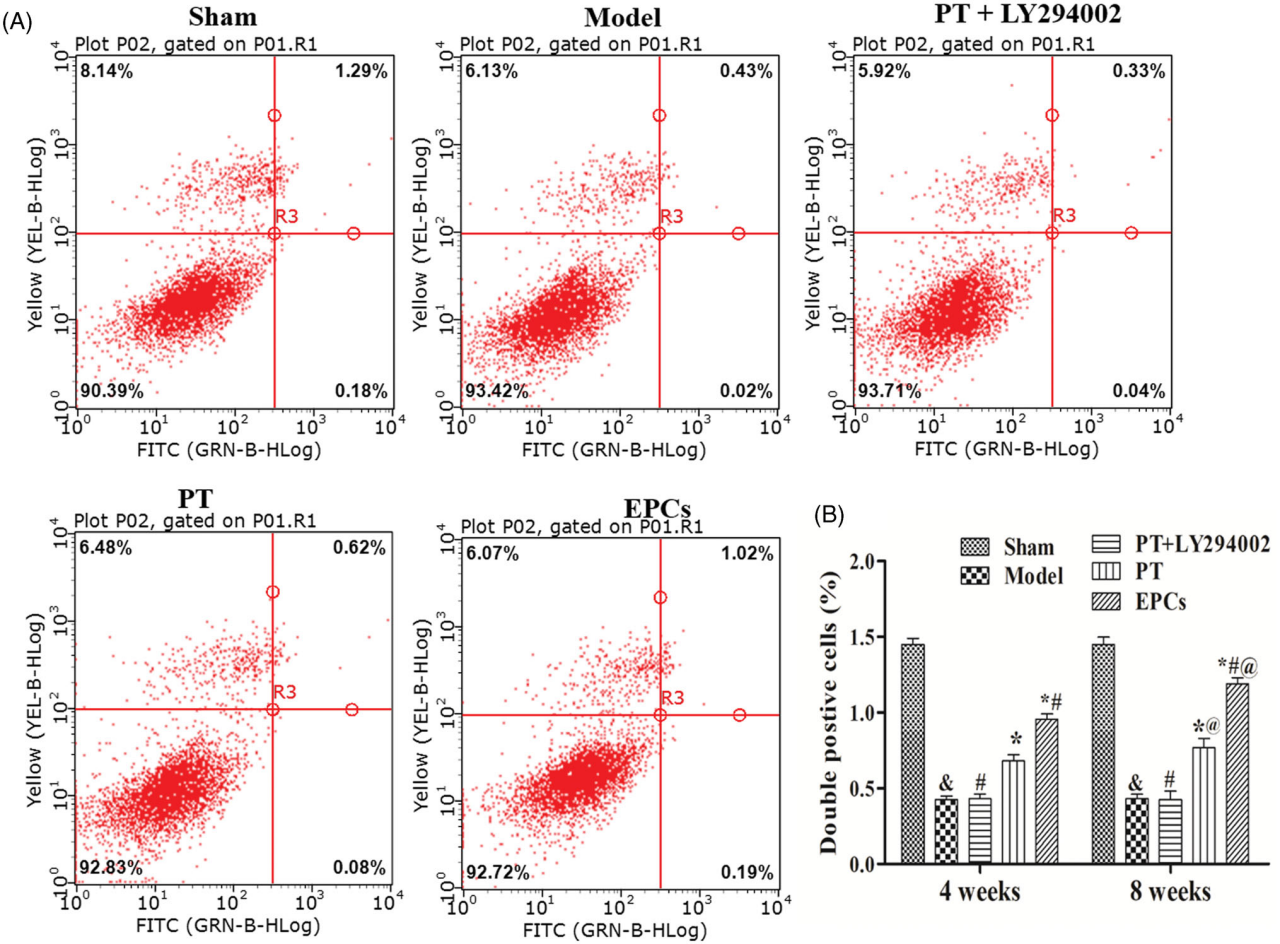
Effect of *PT* on the EPC ratio at 4 weeks and 8 weeks. (A) Flow cytometry images of EPCs at 8 weeks. *X* axis: FITC-UEA. *Y* axis: Dil-acLDL. (B) Statistical evaluation of the EPC ratio at 4 weeks and 8 weeks in each group. The data are presented as the mean ± SD. At 4 weeks and 8 weeks: compared to the sham group, &*p* < 0.05; compared to the model group, *p < 0.05; compared to the *PT* group, #*p* < 0.05; compared to the corresponding 4-week group, @*p* < 0.05.

Flow cytometry (Figure 4) showed that after the rats had been treated for 4 or 8 weeks, the proportion of Dil-acLDL and FITC-UEA double-positive EPCs was significantly greater in the *PT* group than in the model group (*p* < 0.05), while the proportion in the EPC group was significantly greater than that in the *PT* group (*p* < 0.05). Model rats cotreated with *PT* and LY294002 (a PI3K/Akt specific inhibitor) exhibited a significantly smaller proportion of EPCs than rats treated with *PT* alone (*p* < 0.05). These results indicate that *PT* increases the proportion of EPCs in atherosclerotic carotid arteries via the PI3K/Akt pathway.

### PT improves blood lipid levels and inhibits the inflammatory response

We used biochemical detection kits to explore the effects of *PT* on the levels of TCHO, TG, HDL-C and LDL-C in blood. The results (Table 1) showed that the levels of TCHO, TG and LDL-C were significantly higher, while those of HDL-C were lower, in the model group than in the sham group (*p* < 0.05). Compared with the model group, the *PT* group showed decreased TCHO, TG and LDL-C levels in blood and increased HDL-C levels (*p* < 0.05). However, the EPC treatment group did not exhibit different TCHO, TG, HDL-C and LDL-C levels than the model group. In addition, *PT*+LY294002 treatment regulated blood lipid levels only to a limited extent (not as well as *PT* alone).

**Table 1.** Comparison of TCHO, TG, HDL-C and LDL-C at 4 weeks and 8 weeks (*n* = 8).

Groups	Weeks	TCHO (mmol/L)	TG (mmol/L)	HDL-C (mmol/L)	LDL-C (mmol/L)
Sham	4 weeks	1.13 ± 0.22	0.63 ± 0.11	0.67 ± 0.13	0.41 ± 0.12
	8 weeks	1.16 ± 0.20	0.65 ± 0.15	0.68 ± 0.14	0.43 ± 0.13
Model	4 weeks	2.24 ± 0.25^&^	1.27 ± 0.24^&^	0.32 ± 0.086^&^	1.72 ± 0.21^&^
	8 weeks	2.55 ± 0.26^&^	1.35 ± 0.27^&^	0.26 ± 0.075^&^	1.95 ± 0.30^&^
*PT*+LY294002	4 weeks	1.95 ± 0.19^#^	1.18 ± 0.12^#^	0.33 ± 0.10^#^	1.56 ± 0.27^*#^
	8 weeks	2.22 ± 0.32^*#^	1.28 ± 0.26^#^	0.28 ± 0.12^#^	1.76 ± 0.33^#^
*PT*	4 weeks	1.73 ± 0.29*	0.86 ± 0.20*	0.40 ± 0.12*	0.93 ± 0.14*
	8 weeks	1.46 ± 0.26^*@^	0.70 ± 0.15^*@^	0.56 ± 0.13^*@^	0.70 ± 0.25^*@^
EPCs	4 weeks	2.15 ± 0.23^#^	1.28 ± 0.20^#^	0.33 ± 0.096^#^	1.75 ± 0.31^#^
	8 weeks	2.20 ± 0.30^#^	1.40 ± 0.26^#^	0.31 ± 0.11^#^	1.96 ± 0.32^#^

Data are presented as mean ± SD. At 4 weeks and 8 weeks, compared to the sham group; ^&^*p*< 0.05. Compared to the model group; *p< 0.05. Compared to the *PT* group, ^#^*p*< 0.05. Compared to the corresponding 4 weeks’ group, ^@^*p*< 0.05.

ELISAs (Table 2) showed that the concentrations of IL-6 and TNF-α were significantly higher, while the concentration of IL-10 was significantly lower, in the model group than in the sham group. After *PT* treatment, the concentrations of IL-6 and TNF-α were significantly decreased, and that of IL-10 was significantly increased. However, compared with the model group, the EPC treatment group did not exhibit altered IL-6, IL-10 and TNF-α levels. *PT*+LY294002 treatment regulated cytokine levels only to a limited extent (not as well as *PT* alone).

**Table 2.** Comparison of IL-6, IL-10 and TNF-α at 4 weeks and 8 weeks (*n* = 8).

Groups	Weeks	IL-6 (pg/mL)	IL-10 (pg/mL)	TNF-α (pg/mL)
Sham	4 weeks	56.23 ± 5.21	85.62 ± 10.12	86.76 ± 13.18
	8 weeks	54.72 ± 6.34	84.67 ± 11.23	88.57 ± 12.64
Model	4 weeks	102.45 ± 16.25&	31.25 ± 5.23&	226.72 ± 20.11&
	8 weeks	110.52 ± 14.75&	30.21 ± 4.67&	235.53 ± 32.42&
*PT*+LY294002	4 weeks	91.95 ± 13.19	36.98 ± 6.12	200.86 ± 16.17#
	8 weeks	86.48 ± 10.24*#	40.62 ± 7.54*#	189.68 ± 22.86*#
*PT*	4 weeks	81.83 ± 9.22*	40.78 ± 6.09*	164.93 ± 20.14*
	8 weeks	70.26 ± 8.25*@	62.54 ± 8.55*@	135.62 ± 16.42*@
EPCs	4 weeks	101.56 ± 12.31#	28.82 ± 5.10#	216.45 ± 26.14#
	8 weeks	106.59 ± 17.48#	30.12 ± 6.53#	208.63 ± 30.27#

Data are presented as mean ± SD. At 4 weeks and 8 weeks, compared to the sham group; &*p*< 0.05. Compared to the model group; *p< 0.05. Compared to the *PT* group, #*p*< 0.05. Compared to the corresponding 4 weeks’ group, @*p*< 0.05.

These data show that *PT* improves blood lipid levels and inhibits the inflammatory response *in vivo*.

## Discussion

Within 6 months of wire angioplasty and stenting, approximately 15–40% of treated patients present with clinically significant renarrowing of the arteries causing vasospasm, thrombosis and intimal hyperplasia (Swanson et al. 2003). Although the use of stents reduces the incidence of restenosis after angioplasty, intimal hyperplasia, as the main mechanism of poststent restenosis, causes restenosis to remain a substantial clinical problem (Bhardwaj et al. 2005). The roles of VEGFs in intimal hyperplasia and atherogenesis are still unclear. Some studies have reported that certain members of the VEGF family can reduce intimal hyperplasia, while others accelerate restenosis and atherosclerosis (Cooney et al. 2007). In a rabbit model, Bhardwaj et al. (2005) found that efficient adventitial production of VEGF-A and VEGF-D could cause thickening of the inner layer of the artery. Our study found that VEGF expression levels were decreased in intimal hyperplasia model rats, that *PT* treatment upregulated VEGF expression, and that the PI3K/Akt pathway inhibitor LY294002 reduced VEGF expression. These results show that VEGF reduces intimal hyperplasi.

eNOS is an enzyme that catalyses the synthesis of NO. NO-mediated vasodilatory dysfunction and increased cell proliferation occur in vein grafts after surgery, and these pathophysiological changes cause intimal thickening (Sharif et al. 2008). Schwartz (1999) and Sugimoto et al. (2009) revealed that statins upregulate eNOS expression to suppress intimal hyperplasia through Rho-kinase inhibition and that eNOS inhibits intimal hyperplasia and cell proliferation. Overexpression of eNOS can enhance endothelial regeneration and reduce neointimal formation in the vasculature, which may be a promising method for preventing in-stent restenosis and thrombosis (Kawamoto et al. 2003). Our study found that eNOS expression levels were decreased in intimal hyperplasia model rats, that *PT* treatment upregulated eNOS expression, and that the PI3K/Akt pathway inhibitor LY294002 reduced eNOS expression.

The pathogenesis of intimal hyperplasia after vascular injury is thought to involve different signalling cascades that eventually converge on vascular smooth muscle cells (VSMCs), stimulating their proliferation and migration and enhancing the secretion of extracellular matrix (Asahara et al. 1999). Bäck et al. (2005) reported that inhibition of leukotriene B4 and the signalling of its receptor reduces intimal hyperplasia during the response to vascular injury. In vascular ECs, tangerine peels and *PT* tubers may exert therapeutic effects in the treatment of carotid atherosclerosis by upregulating the expression of PBK and p-Akt (Chen et al. 2014). Guo et al. (2017) found that Gualou Xiebai Banxia decoction plays a role in atherosclerotic Apo-E^–/–^ mouse lesions, and the mechanism may be related to the regulation of aortic ICAM-1 and VCAM-1 protein expression. In this study, we found that the PI3K/Akt pathway inhibitor LY294002 weakened the *PT*-mediated improvement in endometrial hyperplasia, reversed the regulatory effect of *PT* on VEGF and eNOS expression and inhibited the increase in the proportion of EPCs. Therefore, we infer that *PT* may regulate intimal hyperplasia in wire-injured atherosclerotic rat carotid arteries via the PI3K/Akt pathway.

Under normal physiological conditions, EPCs are present in the bone marrow. When tissue injury or ischaemia occurs, EPCs migrate to the peripheral blood, specifically migrate to new blood vessels and differentiate into mature ECs (Werner et al. 2005). The discovery of the role of established EPCs in the physiological response to ischaemia has led to the development of new angiogenesis and vascular regeneration strategies (Assmus et al. 2002; Fadini et al. 2007). In the context of human acute myocardial infarction, intracoronary injection of EPCs can improve left ventricular function (Nam et al. 2009). In asymptomatic subjects, a lower number of EPCs is associated with more severe atherosclerosis (Kobayashi et al. 2008). In our study, a flow cytometry assay revealed that *PT* increased the proportions of EPCs in atherosclerotic carotid arteries, indicating that *PT* promoted EPC migration to the peripheral blood from bone marrow. In addition, the PI3K/Akt pathway inhibitor LY294002 blocked EPC migration. Based on these findings, we deduced that *PT* promotes the mobilization and homing of EPCs via the PI3K/Akt pathway.

The occurrence of atherosclerosis is often accompanied by high blood lipid levels and an inflammatory response; thus, lowering the levels of blood lipids and inflammatory cytokines can effectively delay the progression of atherosclerosis (Eswar 2010; Pfeiler and Gerdes 2018). Researchers have found that a variety of natural substances have protective effects against atherosclerosis (Zhang 2019a). For example, Danggui-Buxue-Tang can decrease the concentrations of C-reactive protein (CRP) and TNF-α, increase the survival rate and reduce body weight loss in the context of diabetic atherosclerosis (Zhang et al. 2006). In addition, Gualou Xiebai Banxia decoction can inhibit inflammatory cytokines and regulate lipid metabolism in atherosclerotic Apo-E^–/–^ mouse lesions (Guo et al. 2017). Numerous clinical and experimental studies have confirmed that the traditional Chinese medicine *PT* has protective effects against atherosclerosis (Wang et al. 2008; Chen et al. 2014; Yu et al. 2015; Li et al. 2016; Guo et al. 2017; Zhang 2019b). In this study, we found that *PT* could decrease blood lipid levels and inhibit inflammatory cytokines, whereas EPCs did not regulate blood lipids or the inflammatory response. This study confirms that traditional Chinese medicines such as *PT* have a variety of pharmacological activities.

## Conclusions

This study found that *PT* attenuates intimal hyperplasia in wire-injured atherosclerotic rat carotid arteries via the PI3K/Akt pathway and that this effect may be related to the roles of *PT* in improving blood lipids, inhibiting the inflammatory response and increasing the EPC ratio. Next, our team will further study the effects of *PT* on intimal hyperplasia in wire-injured atherosclerosis patients and explore in depth some other relevant molecular mechanisms, such as those mediated by VSMCs and other signalling pathways.

## Supplementary Material

Supplementary Figure 2

Supplementary Figure 1

## References

[CIT0001] Asahara T, Masuda H, Takahashi T, Kalka C, Pastore C, Silver M, Kearne M, Magner M, Isner JM. 1999. Bone marrow origin of endothelial progenitor cells responsible for postnatal vasculogenesis in physiological and pathological neovascularization. Circ Res. 85(3):221–228. 10436164 10.1161/01.res.85.3.221

[CIT0002] Assmus B, Schächinger V, Teupe C, Britten M, Lehmann R, Döbert N, Grünwald F, Aicher A, Urbich C, Martin H, et al. 2002. Transplantation of progenitor cells and regeneration enhancement in acute myocardial infarction (TOPCARE-AMI). Circulation. 106(24):3009–3017. 12473544 10.1161/01.cir.0000043246.74879.cd

[CIT0003] Bäck M, Bu DX, Bränström R, Sheikine Y, Yan ZQ, Hansson GK. 2005. Leukotriene B4 signaling through NF-kappaB-dependent BLT1 receptors on vascular smooth muscle cells in atherosclerosis and intimal hyperplasia. Proc Natl Acad Sci USA. 102(48):17501–17506. 16293697 10.1073/pnas.0505845102 PMC1297663

[CIT0004] Bhardwaj S, Roy H, Heikura T, Ylä-Herttuala S. 2005. VEGF-A, VEGF-D and VEGF-D^ΔNΔC^ induced intimal hyperplasia in carotid arteries. Eur J Clin Invest. 35(11):669–676. 16269016 10.1111/j.1365-2362.2005.01555.x

[CIT0005] Chen WQ, Huang XB, Wang NQ, Chen YJ. 2014. Effect of paired using tangerine peel and ternate *Pinellia* tuber on the expressions of phosphatidylinositol 3-kinase and phosphorylation of protein kinase B/Akt in rabbits with carotid atherosclerosis. Chin J Cerebrovasc Dis. 11:364–367.

[CIT0006] Cooney R, Hynes SO, Sharif F, Howard L, O'Brien T. 2007. Effect of gene delivery of NOS isoforms on intimal hyperplasia and endothelial regeneration after balloon injury. Gene Ther. 14(5):396–404. 17080182 10.1038/sj.gt.3302882

[CIT0007] Eldika N, Yerra L, Chi DS, Krishnaswamy G. 2004. Atherosclerosis as an inflammatory disease: implications for therapy. Front Biosci. 9:2764–2777. 15353312 10.2741/1434

[CIT0008] Eswar K. 2010. Inflammation, oxidative stress and lipids: the risk triad for atherosclerosis in gout. Rheumatology (Oxford). 49:1229–1238. 20202928 10.1093/rheumatology/keq037

[CIT0009] Fadini GP, Agostini C, Sartore S, Avogaro A. 2007. Endothelial progenitor cells in the natural history of atherosclerosis. Atherosclerosis. 194(1):46–54. 17493626 10.1016/j.atherosclerosis.2007.03.046

[CIT0010] Guo JE, Gao F, Hu YT, Wu JY, Zhang SF, Dong JM, Li JH. 2017. Effects of Gualou Xiebai Banxia decoction on inflammatory cytokines and the expression of ICAM-1 and VCAM-1 in AS model mice. J Jinan Univ. 38:234–239.

[CIT0011] Hoffmann R, Mintz GS. 2000. Coronary in-stent restenosis—predictors, treatment and prevention. Eur Heart J. 21(21):1739–1749. 11052838 10.1053/euhj.2000.2153

[CIT0012] Kawamoto A, Tkebuchava T, Yamaguchi J, Nishimura H, Yoon YS, Milliken C, Uchida S, Masuo O, Iwaguro H, Ma H, et al. 2003. Intramyocardial transplantation of autologous endothelial progenitor cells for therapeutic neovascularization of myocardial ischemia. Circulation. 107(3):461–468. 12551872 10.1161/01.cir.0000046450.89986.50

[CIT0013] Kobayashi H, Shimizu T, Yamato M, Tono K, Masuda H, Asahara T, Kasanuki H, Okano T. 2008. Fibroblast sheets co-cultured with endothelial progenitor cells improve cardiac function of infarcted hearts. J Artif Organs. 11(3):141–147. 18836875 10.1007/s10047-008-0421-8

[CIT0014] Lefkovits J, Topol EJ. 1997. Pharmacological approaches for the prevention of restenosis after percutaneous coronary intervention. Prog Cardiovasc Dis. 40(2):141–158. 9327830 10.1016/s0033-0620(97)80006-0

[CIT0015] Li Y, Li D, Chen J, Wang S. 2016. A polysaccharide from *Pinellia ternata* inhibits cell proliferation and metastasis in human cholangiocarcinoma cells by targeting of Cdc42 and 67 kDa laminin receptor (LR). Int J Biol Macromol. 93(Pt A):520–525. 27576948 10.1016/j.ijbiomac.2016.08.069

[CIT0016] Nam D, Ni CW, Rezvan A, Suo J, Budzyn K, Llanos A, Harrison D, Giddens D, Jo H. 2009. Partial carotid ligation is a model of acutely induced disturbed flow, leading to rapid endothelial dysfunction and atherosclerosis. Am J Physiol Heart Circ Physiol. 297(4):H1535–H1543. 19684185 10.1152/ajpheart.00510.2009 PMC2770764

[CIT0017] Pfeiler S, Gerdes N. 2018. Atherosclerosis: cell biology and lipoproteins – focus on anti-inflammatory therapies. Curr Opin Lipidol. 29(1):53–55. 29298275 10.1097/MOL.0000000000000481

[CIT0018] Schwartz SM. 1999. The intima: a new soil. Circ Res. 85(10):877–879. 10559132 10.1161/01.res.85.10.877

[CIT0019] Sharif F, Hynes SO, Cooney R, Howard L, McMahon J, Daly K, Crowley J, Barry F, O'Brien T. 2008. Gene-eluting stents: adenovirus-mediated delivery of eNOS to the blood vessel wall accelerates re-endothelialization and inhibits restenosis. Mol Ther. 16(10):1674–1680. 18714308 10.1038/mt.2008.165

[CIT0020] Sugimoto M, Dai Y, Komori K. 2009. Therapeutic approach against intimal hyperplasia of vein grafts through endothelial nitric oxide synthase/nitric oxide (eNOS/NO) and the Rho/Rho-kinase pathway. Surg Today. 39(6):459–465. 19468800 10.1007/s00595-008-3912-6

[CIT0021] Swanson N, Hogrefe K, Javed Q, Malik N, Gershlick AH. 2003. Vascular endothelial growth factor (VEGF)-eluting stents: *in vivo* effects on thrombosis, endothelialization and intimal hyperplasia. J Invasive Cardiol. 15(12):688–692. 14660819

[CIT0022] Vasa M, Fichtlscherer S, Aicher A, Adler K, Urbich C, Martin H, Zeiher AM, Dimmeler S. 2001. Number and migratory activity of circulating endothelial progenitor cells inversely correlate with risk factors for coronary artery disease. Circ Res. 89(1):E1–E7. 11440984 10.1161/hh1301.093953

[CIT0023] Wang LL, Gao L, Zheng SX, Zhou FJ. 2008. Clinical observation of hyperlipemia treated with different *Pinellia* products in Gualou Xiebai Banxia Tang. World J Integ Trad West Med. 9:542–543.

[CIT0024] Werner N, Kosiol S, Schiegl T, Ahlers P, Walenta K, Link A, Böhm M, Nickenig G. 2005. Circulating endothelial progenitor cells and cardiovascular outcomes. Acc Curr J Rev. 14:999–1007. 10.1056/NEJMoa043814 16148285

[CIT0025] Yasukawa H, Imaizumi T, Matsuoka H, Nakashima A, Morimatsu M. 1997. Inhibition of intimal hyperplasia after balloon injury by antibodies to intercellular adhesion molecule-1 and lymphocyte function-associated antigen-1. Circulation. 95(6):1515–1522. 9118520 10.1161/01.cir.95.6.1515

[CIT0026] Yoshimura S, Morishita R, Hayashi K, Yamamoto K, Nakagami H, Kaneda Y, Sakai N, Ogihara T. 2001. Inhibition of intimal hyperplasia after balloon injury in rat carotid artery model using cis-element 'decoy' of nuclear factor-kappaB binding site as a novel molecular strategy. Gene Ther. 8(21):1635–1642. 11895002 10.1038/sj.gt.3301566

[CIT0027] Young M, Cuculi F, Erne P. 2013. PTCA with drug-coated balloons is associated with immediate decrease of coronary flow reserve. Catheter Cardiovasc Interv. 81(4):682–686. 23361864 10.1002/ccd.23502

[CIT0028] Yu HL, Zhao TF, Wu H, Pan YZ, Zhang Q, Wang KL, Zhang CC, Jin YP. 2015. *Pinellia ternata* lectin exerts a pro-inflammatory effect on macrophages by inducing the release of pro-inflammatory cytokines, the activation of the nuclear factor-κB signaling pathway and the overproduction of reactive oxygen species. Int J Mol Med. 36(4):1127–1135. 26310942 10.3892/ijmm.2015.2315

[CIT0029] Zhang G, Jiang N, Song W, Ma CH, Yang SC, Chen JW. 2016. *De novo* sequencing and transcriptome analysis of *Pinellia ternata* identify the candidate genes involved in the biosynthesis of benzoic acid and ephedrine. Front Plant Sci. 7:1–14. 27579029 10.3389/fpls.2016.01209 PMC4986801

[CIT0030] Zhang H, Chen S, Deng X, Yang X, Huang X. 2006. Danggui-Buxue-Tang decoction has an anti-inflammatory effect in diabetic atherosclerosis rat model. Diabetes Res Clin Pract. 74(2):194–196. 16713007 10.1016/j.diabres.2006.04.003

[CIT0031] Zhang J. 2019a. Clinical observation on treating angina pectoris from coronary atherosclerotic heart disease with the Gualou Xiebai decoction. Clin J Chin Med. 11:23–25.

[CIT0032] Zhang Y. 2019b. New evidence of anti-atherosclerosis in traditional Chinese medicine. Chin J Cardiol. 47:339–340.

[CIT0033] Zoldhelyi P, Chen ZQ, Shelat HS, McNatt JM, Willerson JT. 2001. Local gene transfer of tissue factor pathway inhibitor regulates intimal hyperplasia in atherosclerotic arteries. Proc Natl Acad Sci USA. 98(7):4078–4083. 11274432 10.1073/pnas.061004098 PMC31182

