# Supplementary figures and images for: Pinellia ternata attenuates carotid artery intimal hyperplasia and increases endothelial progenitor cell activity via the PI3K/Akt signalling pathway in wire-injured rats
Source: Pharm Biol. 2020 Nov 30;58(1):1193–200. doi: 10.1080/13880209.2020.1845748 (PMC7717851; doi:10.1080/13880209.2020.1845748)

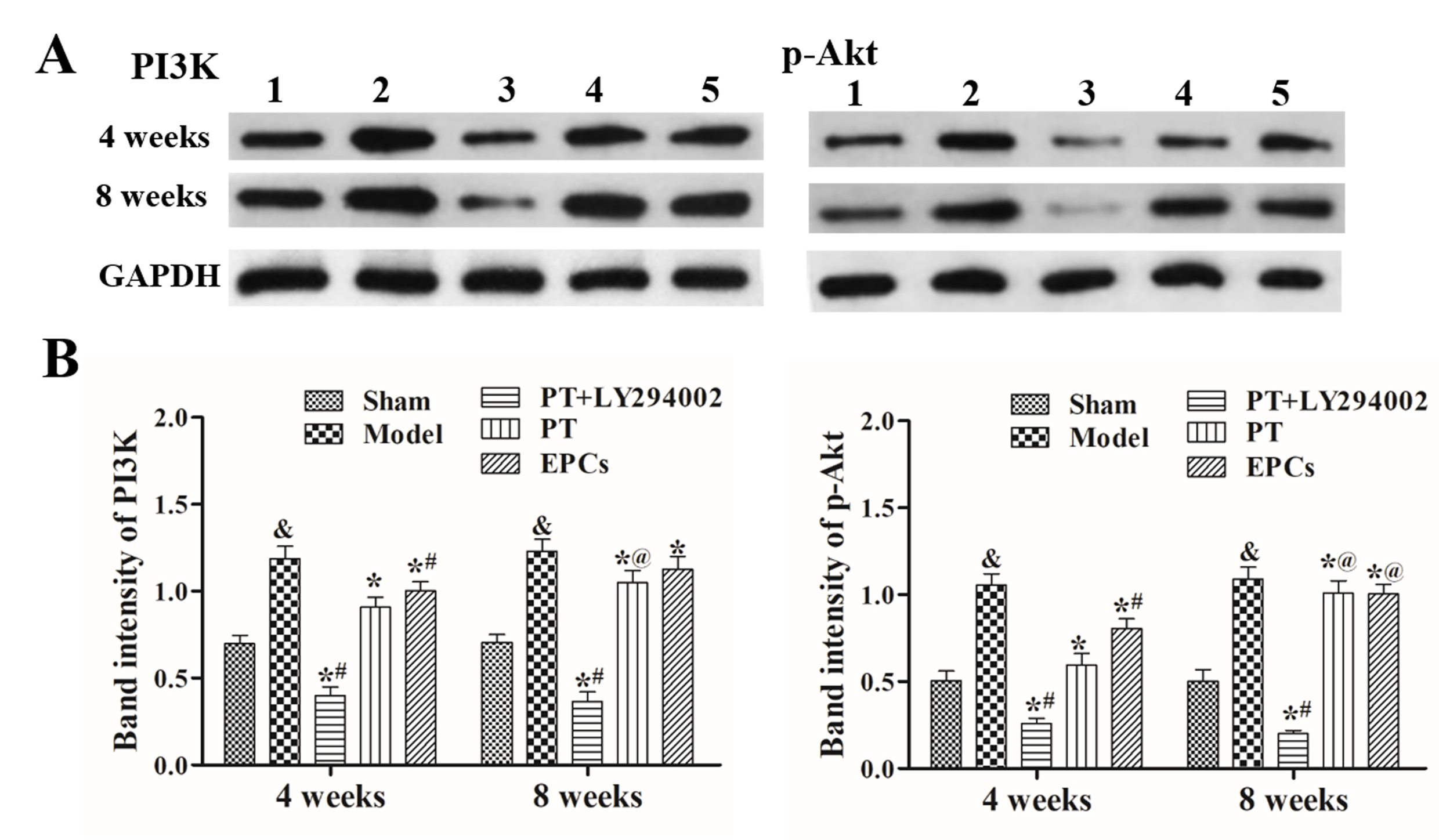

Supplement: Supplementary Figure 2 [file IPHB_A_1845748_SM5744.tif]

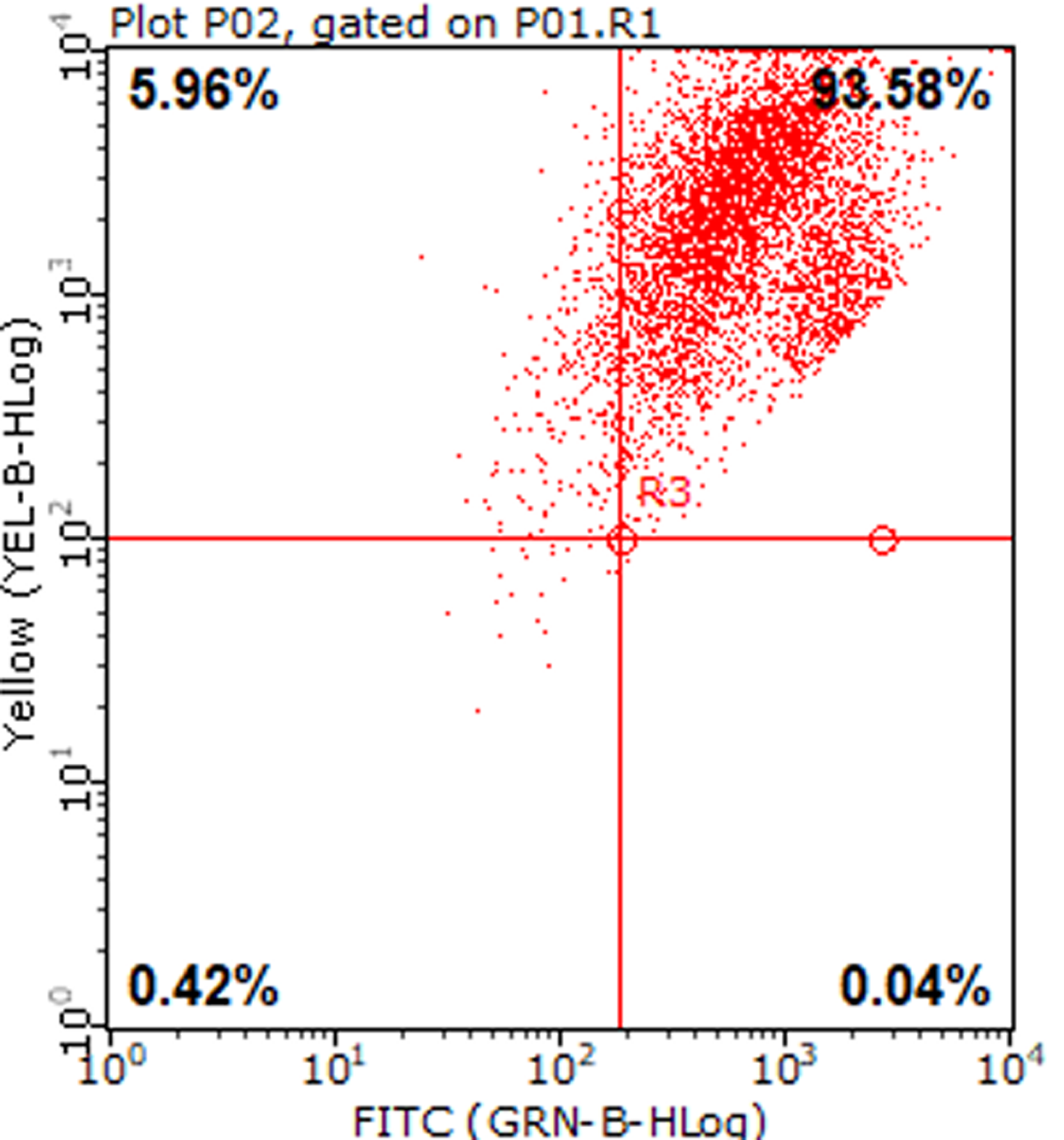

Supplement: Supplementary Figure 1 [file IPHB_A_1845748_SM5743.tif]
